# Therapeutic potential of targeting the receptor for advanced glycation end products (RAGE) by small molecule inhibitors

**DOI:** 10.1002/ddr.21971

**Published:** 2022-07-04

**Authors:** Harbinder Singh, Devendra K. Agrawal

**Affiliations:** ^1^ Department of Translational Research College of Osteopathic Medicine of the Pacific Western University of Health Sciences Pomona California USA

**Keywords:** AGEs, antagonist, chronic inflammatory diseases, endogenous ligands, exogenous ligands, inhibitor, RAGE, RAGE isoforms, S100 proteins

## Abstract

Receptor for advanced glycation end products (RAGE) is a 45 kDa transmembrane receptor of immunoglobulin family that can bind to various endogenous and exogenous ligands and initiate the inflammatory downstream signaling pathways. RAGE is involved in various disorders including cardiovascular and neurodegenerative diseases, cancer, and diabetes. This review summarizes the structural features of RAGE and its various isoforms along with their pathological effects. Mainly, the article emphasized on the translational significance of antagonizing the interactions of RAGE with its ligands using small molecules reported in the last 5 years and discusses future approaches that could be employed to block the interactions in the treatment of chronic inflammatory ailments. The RAGE inhibitors described in this article could prove as a powerful approach in the management of immune‐inflammatory diseases. A critical review of the literature suggests that there is a dire need to dive deeper into the molecular mechanism of action to resolve critical issues that must be addressed to understand RAGE‐targeting therapy and long‐term blockade of RAGE in human diseases.

## 1 INTRODUCTION

**Figure 1:**
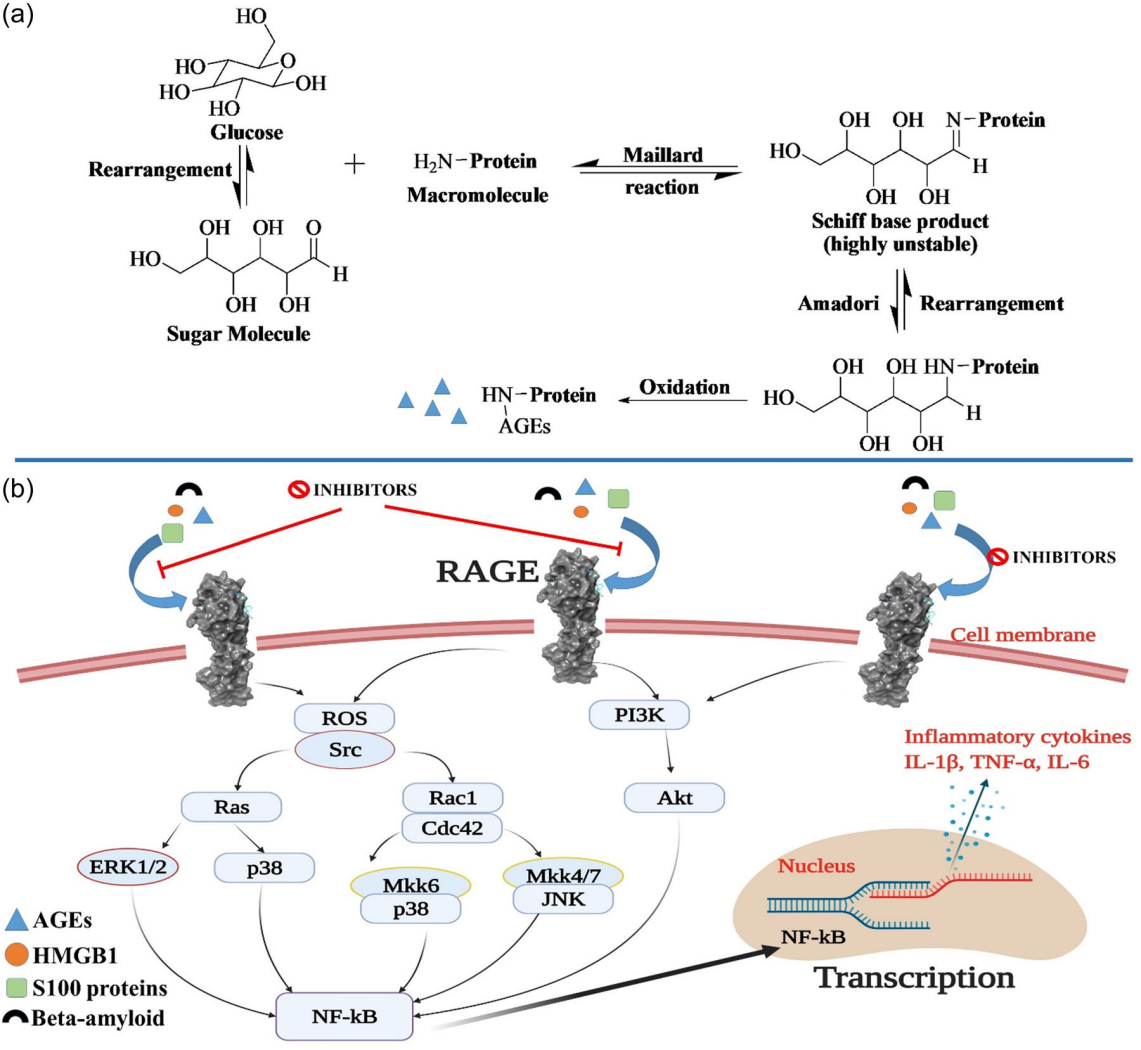
(a) Mechanisms of glycation to produce AGEs and (b) Ligand binding with RAGE and the downstream signaling pathways. AGEs, advanced glycation end products; RAGE, receptor for advanced glycation end products

Various sugar molecules, such as glyceraldehyde, glucose, and fructose, nonenzymatically can react with amine‐based macromolecules such as proteins, nucleic acids, and lipids to produce reversible Schiff bases. These Schiff bases further undergo various complex reactions like rearrangement, dehydration, and condensation to form irreversible adduct known as advanced glycation end products (AGEs) (Akhter et al., 2015; Jabir et al., 2018; Yamagishi, 2011). Nonenzymatic glycation of macromolecules alters their physiological function and structural integrity that ultimately leads to loss of enzymatic function, protein aggregation, and cross‐linking (Adrover et al., 2014; Li et al., 2012; Nenna et al., 2015; Sajithlal et al., 1998; Vistoli et al., 2013). The accumulation of the advanced glycation products (AGEs) plays a significant role in many inflammatory health disorders including cardiovascular, diabetes mellitus, immune‐inflammation, cancer, and neurodegenerative disorders (Li et al., 2012; Logsdon et al., 2007; Nenna et al., 2015; Ramasamy et al., 2005; Ray et al., 2016; Sparvero et al., 2009). Functionally, AGEs are recognized by the cell surface receptor of immunoglobulin superfamily called as receptor for advanced glycation end products (RAGE). RAGE is a 45 kDa transmembrane receptor that is present in very low concentration in healthy human tissues such as liver, kidneys, lungs, brain, cardiovascular, and immune systems (Cheng et al., 2005; Neeper et al., 1992). In addition to AGEs, RAGE can interact with a number of endogenous ligands including S100/calgranulin proteins, HMGB1 (high mobility group box‐1), Aβ_1‐42_‐peptides (amyloid‐β), and exogenous ligand, LPS (lipopolysaccharides) (Chavakis et al., 2003; Fritz, 2011; Hudson & Lippman, 2018). As a receptor for these ligands, RAGE itself has been considered as a potential biomarker for various pathological conditions like cardiovascular diseases, diabetes mellitus, diabetic nephropathy, cancer, and Alzheimer's disease (Deane et al., 2012; Logsdon et al., 2007; Nabi et al., 2019; Nasser et al., 2015; Ramasamy et al., 2005; Ray et al., 2016; Riehl et al., 2009; Tabrez et al., 2015). The overall mechanisms of glycation to produce AGEs and various ligand binding with RAGE to overexpress the inflammatory cytokines are depicted in Figure 1a,b, respectively.

Expression of RAGE has been found in many embryonic tissues but its expression decreases in adult tissues, except lung and skin (Bierhaus et al., 2005). Its high expression has been found in basolateral membrane of epithelial cells AT‐1, and AT‐2 cells, alveolar macrophages, and bronchiolar epithelia (Katsuoka et al., 1997; Oczypok et al., 2017). Under inflammatory conditions, RAGE expression is significantly upregulated in neuronal cells, vascular smooth muscle cells (VSMCs), endothelial cells (ECs), airway smooth muscle cells, pericytes, and in circulating eosinophils, dendritic cells, macrophages, T cells, and B cells (Bierhaus et al., 2005; Brett et al., 1993; Chuah et al., 2013; Hudson & Lippman, 2018; Lin et al., 2009; Yonekura et al., 2003).

**Figure 2:**
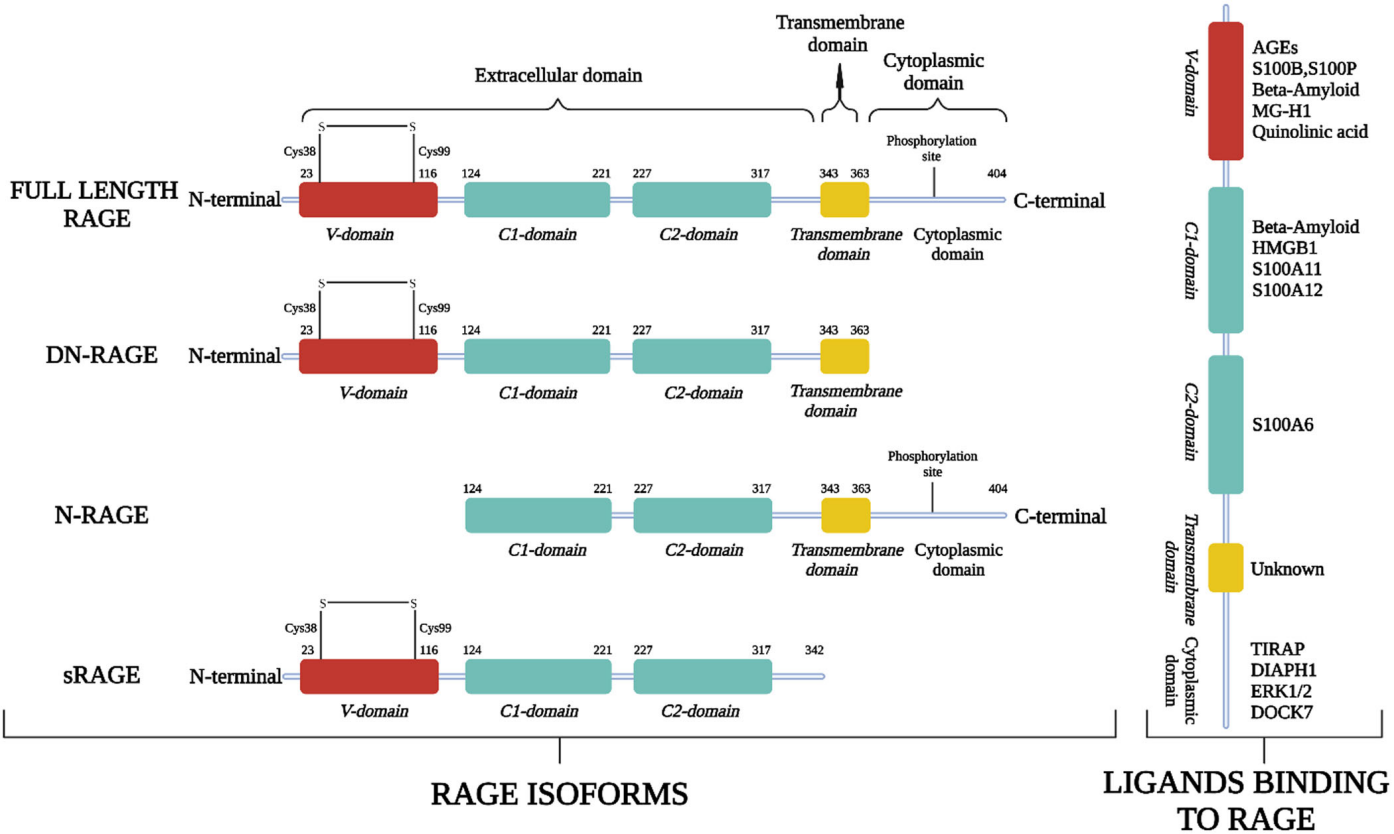
(Left) Full‐length RAGE and its various isoforms: Full‐length RAGE consists of Variable domain (V‐domain), two constant domains (C1 and C2), transmembrane domain, and cytoplasmic domain; its isoforms dominant negative RAGE (DN‐RAGE), N‐truncated RAGE (N‐RAGE), and secretory or soluble RAGE (sRAGE) with their respective domains. (Right) Various extracellular and intracellular ligands binding with RAGE. RAGE, receptor for advanced glycation end products

Structurally, full‐length human RAGE is a 45 kDa protein that consists of three major domains: First, the extracellular domain with 23–342 amino acid residues, second is hydrophobic transmembrane domain with 343–363 amino acid residues, and third one is intracellular cytoplasmic domain with 464–404 residues (UniProtBeta, 2022) (Figure 2). An extracellular region further subdivided into three immunoglobulin like domains named: Variable domain (V‐domain) having 23–116 amino acid residues which is connected to two constant domains C1 (residues 124–221) and C2 (residues 227–317). Various strands present in the V‐domain of RAGE are connected through two *β*‐sheets which are linked together by a disulfide bridge between two cysteine amino acid residues (Cys38 and Cys99) (Xue et al., 2011; Yatime & Andersen, 2013) (Figure 2). The surface around V‐domain and C1 domain is covered by a large positively charged area and a hydrophobic cavity. Various studies demonstrated that the integrated structural unit of V and C1 domain is primarily responsible for the interactions with a diverse group of RAGE ligands of negatively charged molecules, including S100/calgranulins, AGEs, HMGB1, and A*β*‐proteins to exert their specific effects (Dattilo et al., 2007; Hori et al., 1995; Koch et al., 2010; Sturchler et al., 2008; Xue et al., 2011, 2014).

RAGE may undergo ligand‐driven dimerization or oligomerization, the stability of which might provide an explanation for the affinity or specificity of RAGE towards the ligands and its resulting signal transduction (Xie et al., 2007, 2008; Xu et al., 2013; Zong et al., 2010). Its C2 domain is an independent structural unit that is connected to C1 domain via a linker of 12 amino acid residues (Dattilo et al., 2007). Similar to the VC1 structural unit, C2 also has a large negatively charged surface covered by acidic amino acid residues directed toward the surface of VC1 oligomer (Yatime & Andersen, 2013). The extracellular domain of human RAGE has more than 75% structural similarity (sequence identity) with mice, rats, and primates (Rodriguez Gonzalez‐Moro et al., 2009). In all these species, the amino acid residues involved in the binding of AGE and RAGE are conserved to Lys52, Arg98, and Lys110 indicating a common binding pattern in all four species (Xie et al., 2008; Xue et al., 2011, 2014).

Due to the lack of sufficient data on the transmembrane domain of RAGE (residues 343–363), little is known, so far, about its structure and functions. However, it induces signal transduction probably due to the helix‐helix homodimerization of the receptor (Sturchler et al., 2008).

The cytoplasmic domain of RAGE (residues 364–404) also has three regions: a 17‐amino acid domain known as membrane‐proximal domain, this is further connected to central 17‐amino acid domain, and C‐terminal domain. These domains also perform crucial interactions with various downstream signaling effector molecules such as Toll‐interleukin 1 receptor domain adaptor protein (TIRAP), diaphanous related formin‐1 (DIAPH1), and extracellular signal‐regulated kinases 1 and 2 (ERK1/2) that ultimately lead to the activation of mitogen‐activated protein kinase (MAPK) pathway (Hudson et al., 2008; Ishihara et al., 2003; Rai et al., 2012). This domain of human RAGE also shares 92% and 70% sequence identity with primates and rodents, respectively (Rodriguez Gonzalez‐Moro et al., 2009).

According to the alternative splicing and metalloproteinase‐regulated cleavage, RAGE can exist as multiple variants with different binding partners and can perform diverse biological functions (Jules et al., 2013; Zhang et al., 2008). Figure 2 represents the full‐length RAGE and its various isoforms with their amino acid sequence length. The full‐length RAGE consists of all key constituents with amino acid sequence of 23–404 residues and can perform a key role in diverse downstream signaling pathways that can affect inflammatory responses, oxidative stress, cellular proliferation, migration, and apoptosis (Hong et al., 2016; Hudson et al., 2008; Jules et al., 2013). Deletion of cytoplasmic domain via metalloproteinases generates a dominant‐negative RAGE (DN‐RAGE; residues 23–363) which shows dominant‐negative effect with reduced signaling response to RAGE ligands (Kosaka et al., 2014) (Figure 2). The overexpression of DN‐RAGE can attenuate the cancer cell proliferation and invasion in in vitro and in vivo experiments (Takeuchi et al., 2013). Similarly, lacking the N‐terminal variable domain (V‐domain) in N‐RAGE (residues 124–404) diminished its ability to bind with various ligands that can bind to this domain. However, N‐RAGE can exhibit its V‐domain‐independent pathological functions and signal transduction (Yonekura et al., 2003). Further, the cleavage of the extracellular domain from the cell surface via metalloproteinases produce soluble form of RAGE (sRAGE; residues 23–342) which is the dominant form counteracting the RAGE‐mediated functions by acting as its decoy receptor (Emanuele et al., 2005; Falcone et al., 2005; Kalea et al., 2009; Scavello et al., 2021). The direct administration of sRAGE in vivo has been shown to reverse the RAGE‐mediated pathological conditions (Cho et al., 2009; Geroldi et al., 2006; Kalea et al., 2009; Scavello et al., 2021).

## 2 LIGANDS THAT CAN BIND TO RAGE

Due to the presence of various domains (V, C1, and C2) on RAGE, it can bind to the diverse classes of ligands, including AGEs, S100 calcium‐binding proteins, amyloid‐β, HMGB1, and so forth (Chavakis et al., 2003; Hofmann et al., 1999; Hori et al., 1995; Orlova et al., 2007; Santilli et al., 2009; Schmidt et al., 2000; Yan et al., 1996) (Figure 2). The ligand binding to RAGE activates multiple signaling pathways, such as ERK, STAT3, MAPK, and JNK that result in the augmentation of transcription factors, including nuclear factor kappa B (NF‐kB) (Fritz, 2011; Kierdorf & Fritz, 2013) (Figure 1b). These ligand‐RAGE interactions are primarily involved in the pathogenesis of various inflammatory diseases such as atherosclerosis, diabetes mellitus, cancer, neurodegenerative disorders (Alzheimer's disease), rheumatoid arthritis, and chronic renal failure (Basta, 2008; Hofmann et al., 2002; Ramasamy et al., 2005; Rong et al., 2005; Schmidt et al., 1999; Schmidt et al., 2001; Taguchi et al., 2000; Tanji et al., 2000). Various extracellular and intracellular ligands that can bind to RAGE along with their clinical implications and binding sites on RAGE are summarized in Table 1.

**Table 1:** Various extracellular and intracellular RAGE binding ligands with their clinical implications

Ligand	Type	Binding domain	Binding affinity	Clinical implication	References
AGEs (MG‐H1)	Extracellular ligand	V	ND	Cardiovascular diseases (atherosclerosis), Diabetes, cancer, chronic inflammation	(Heier et al., 2015; Wetzels et al., 2019; Xue et al., 2011; Yamagishi, 2019)
S100B	Extracellular ligand	VC1	3.2–9.4 µM (ITC)	Inflammatory response and Neuronal disease, Down syndrome, Alzheimer's disease.	(Chen et al., 2020; Dattilo et al., 2007; Esposito et al., 2008)
		VC1	*K*_d1_ = 11 nM, *K*_d1_ = 244 nM (SPR)		
		V	*K*_d1_ = 550 nM, *K*_d2_ = 470 nM (SPR)		
S100P	Extracellular ligand	V	6 µM	Cancer disease and chronic inflammation	(Penumutchu et al., 2014), (Lin et al., 2021)
S100A6	Extracellular ligand	VC1	0.6–5.8 µM	Alzheimer's disease (AD), epileptogenesis, amyotrophic lateral sclerosis (ALS), Huntington's disease (HD), and Parkinson's disease (PD)	(Filipek & Lesniak, 2020; Leclerc et al., 2007)
		V	0.5–13.5 µM		
		C2	28 nM to 1 µM		
S100A11	Extracellular ligand	V	0.5 µM (Immunofluorescence assay)	Cancer, Neurological diseases, Vascular calcification, and inflammatory diseases.	(Zhang et al., 2021; Huang et al., 2016)
S100A12	Extracellular ligand	C1	70 nM	Cardiovascular diseases, Inflammatory response, and cancer progression	(Chiou et al., 2016; Saito et al., 2012; Wang et al., 2020; Xie et al., 2007)
		V	3.1 µM (Immunofluorescence assay)		
Amyloid‐β	Extracellular ligand	V & C1	70–80 nM	Neurodegenerative disorders (Alzheimer's disease)	(Deane et al., 2012)
Quinolinic acid	Extracellular ligand	VC1	43 nM	Huntington's disease, hepatic encephalopathy, AIDS‐dementia complex, and Alzheimer's disease	(Schwarcz et al., 2012; Serratos et al., 2015)
HMGB1	Extracellular ligand	VC1C2	6–10 nM	Chronic inflammation and cancer	(Hori et al., 1995; Sims et al., 2010)
DNA/RNA	Extracellular ligand	VC1	ND	Chronic inflammation	(Sirois et al., 2013)
RSV F‐protein	Extracellular ligand	VC1	Very low binding (Alpha Screen assay	Modulates lower respiratory tract infection	(Tian et al., 2013)
Longistatin	Extracellular ligand	V	72 nM	Acts as an antagonist and can reduce the inflammation	(Anisuzzaman et al., 2014)
TIRAP	Intracellular ligand	Cytoplasmic domain	ND	Chronic inflammation	(Rajpoot et al., 2021)
DIAPH1	Intracellular ligand	Cytoplasmic domain	4–8 µM	Diabetes, cardiovascular diseases	(Egana‐Gorrono et al., 2020; Rai et al., 2012)
ERK1/2	Intracellular ligand	Cytoplasmic domain	ND	Cancer and chronic inflammation	(Ishihara et al., 2003)
DOCK7	Intracellular ligand	Cytoplasmic domain	ND	Cancer and chronic inflammation	(Yamamoto et al., 2013)
C1q	Unknown	Unknown	ND	Recruitment of phagocytosis	(Ma et al., 2012)
PS	Unknown	Unknown	ND	Alveolar macrophagic Rac1 activation	(He et al., 2011)

Abbreviation: RAGE, receptor for advanced glycation end products.

## 3 RAGE INHIBITORS

### 3.1 Small inhibitors that bind to extracellular domain of RAGE to inhibit the interactions with its extracellular ligands

The inhibition of ligand binding to RAGE is the best approach to attenuate the pathology of RAGE‐mediated inflammation in various disease conditions. As summarized in Table 1, majority of the ligand activators of RAGE bind to the extracellular domain (V‐domain). Several research groups published synthetic molecules to inhibit the interactions between RAGE and its ligands to treat various disease conditions. Among them, small inhibitors which were published before 2017 have already been summarized (Bongarzone et al., 2017). In this article, we summarized the small RAGE inhibitors published within the period of 2017–2021, along with their pharmacology, structure–activity relationship, and the available information on the binding mechanism with RAGE. Some key molecules having exemplary potential to inhibit RAGE that could treat RAGE‐associated diseases published before 2017 are also described in this article with the new findings.

**Figure 3:**
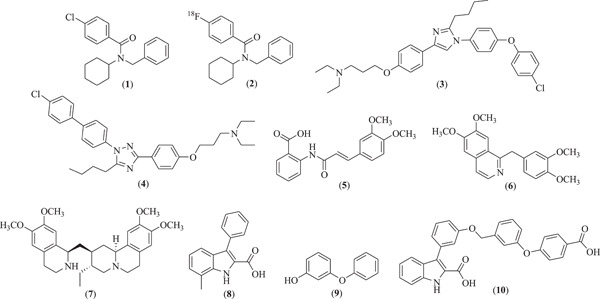
Molecular structure of various RAGE inhibitors that bind to its extracellular domain. RAGE, receptor for advanced glycation end products

A very good example of RAGE inhibitor is FPS‐ZM1 (compound **1**), that was found by screening the 5000 compounds that can block the interactions between RAGE V‐domain and Aβ_1‐42_ to treat Alzheimer's disease (Deane et al., 2012) (Figure 3). It can also inhibit the extracellular domain of RAGE from binding HMGB1 and S100B with the Ki (dissociation constant values) of 148 nM and 230 nM, respectively (Deane et al., 2012). FPS‐ZM1 inhibits the activation of primary microglia by AGEs, and leads to decrease in the expression if RAGE, oxidative stress and thus lowers the level of inflammation (Shen et al., 2017). Recently, it has also been revealed that FPZ‐ZM1 also alleviates the renal injury in hypertensive rats through RAGE inhibition (Liu et al., 2020). FPS‐ZM1 has the perfect structural features that are mainly associated with RAGE binding. Its benzyl group attached directly to the amide nitrogen atom provides the electron‐rich environment to its terminal group (benzyl group). Its tertiary amide central core provides the hydrogen‐bond accepting and donating ability with amino acid residues present within the V‐domain of RAGE. The electron deficient benzene ring attached to carbonyl carbon atom and six‐membered acyclic chain of FPS‐ZM1 provides the stability within the V‐domain of RAGE. Its fluorine‐18 (radiolabeled) analog (compound **2**) was developed for imaging purposes for in vivo studies to trace RAGE which have excellent binding affinity with RAGE V‐domain (*K*_d_ = 15 nM) (Cary et al., 2016) (Figure 3). This compound showed co‐localization with RAGE in the brain samples of Alzheimer's disease when tested through immunohistochemistry. It has superior central nervous system (CNS) penetration and increased uptake in several areas of the brain which are known to express RAGE than its parent analog FPS‐ZM1. The binding modes of compound **2** were streamlined by using docking studies which are stabilized in the V‐domain of RAGE via various hydrophobic interactions with Pro45, Leu49, Trp51, Pro66, Leu78, and Pro80 (Cary et al., 2016).

Another example of RAGE antagonist is the small molecule azeliragon (**3**), also known as PF‐04494700. This molecule has been reported to inhibit the interactions of RAGE with extracellular ligands including S100B, HMGB1, and AGEs (Sabbagh et al., 2011) (Figure 3). Additionally, compound **3** was also found to inhibit the binding of extracellular domain of RAGE to Aβ_1‐42_ with the IC_50_ value of 500 nM which was determined by fluorescence polarization assay (Jones et al., 2012; Mjalli et al., 2009). The compound **3** reduced the neuroinflammation by reducing the level of Aβ_1‐42_ plaque deposition and level of inflammatory cytokines (Burstein et al., 2018). Unfortunately, one clinical study revealed that compound **3** did not appear to exhibit consistent effect on the plasma levels of Aβ_1‐42_ and inflammatory biomarkers (Burstein et al., 2014) and failed in the phase III study to treat the Alzheimer's disease (“2‐Year Extension Study of Azeliragon in Subjects With Alzheimer's Disease (STEADFAST Extension),” 2021; “Evaluation of the Efficacy and Safety of Azeliragon (TTP488) in Patients With Mild Alzheimer's Disease”, 2021). Moreover, the mechanism of binding of compound **3** with RAGE remains ambiguous as no structural data are available to establish their binding modes and locations. However, molecular docking studies performed by Xie et al. provide some basic notions of its binding mode with RAGE V‐domain (Xie et al., 2021) and they used this information to further design novel RAGE inhibitors by modifying the central structural moiety of azeliragon i.e., imidazole to triazole. They synthesized the designed compounds and tested them to check their anticancer potential against triple‐negative breast cancer cell line (TNBC) which is the most aggressive breast cancer cell line. Amongst all the compounds, compound **4** was found to be endowed with the best inhibitory potential against TNBC cells with the IC_50_ value of 0.220 µM (Xie et al., 2021) (Figure 3).

Tranilast (compound **5**) is an anti‐allergic drug molecule that significantly inhibits the binding interactions of V‐domain of RAGE with S100A11 and S100A12 which was tested using proton and nitrogen NMR titrations, fluorescence experiments, and WST‐1 assay (Chiou et al., 2016; Huang et al., 2016) (Figure 3). Different interacting amino acids present in the active domain (V‐domain) of RAGE with tranilast were also revealed using high ambiguity‐driven biomolecular docking (HADDOCK). These results provide the mechanistic details of tranilast which could further be used for the development of novel RAGE inhibitors. Similarly, papaverine (a dimethoxy substituted isoquinoline derivative **6**) was found to significantly inhibit the RAGE‐dependent nuclear factor κ‐B (NF‐κB) activation driven by high mobility group box‐1 (HMGB1) (El‐Far et al., 2018) (Figure 3). C6 glioma cells were used to check the effect of papaverine on HMGB1‐induced NF‐κB activation. Papaverine (compound **6**) at 10 and 20 µM concentration successively inhibited this upregulation. To evaluate the binding mechanism of papaverine with RAGE, AGE‐RAGE binding assay was performed which revealed that the papaverine can bind to the binding site of AGE (i.e., V‐domain of RAGE) (El‐Far et al., 2018). The amino acids of V‐domain of RAGE involved in these interactions are still unknown. Recent studies stated that papaverine also can suppress the chronic inflammatory pain in mice model, while it did not show the anti‐nociceptive effects in the state of oxidative stress at the site of inflammation (Yoshizawa et al., 2021). Therefore, papaverine and its derivatives could act as lead molecules in the development of novel potent RAGE antagonist that can be used to treat various RAGE‐associated diseases.

In 2019, Ahmad et al. (2019) claimed a natural molecule emetine (compound **7**) as a potent inhibitor of RAGE and Aβ_1‐42_ interactions which was confirmed through computational studies (Figure 3). Compound **7** itself can bind efficiently with Aβ_1‐42_ with the calculated energy −6.99 kcal/Mol but was found to interact with the same amino acid residues of Aβ_1‐42_ which are responsible for binding with RAGE V‐domain (Ahmad et al., 2019), which suggests that emetine (compound **7**) could be the novel invention for the further development of anti‐Alzheimer's agents that affect the RAGE signaling pathway. The actual potential of compound **7** to inhibit these interactions are experimentally unproven. Similarly, aminopyrimidine derivatives and phenyl benzoxazoles derivatives discovered a few years back (structures are not provided) were also found to show excellent inhibition in the interactions of RAGE and Aβ_1‐42_ with the potential to treat AD, however the actual binding mechanism with RAGE was not predicted (Choi et al., 2015; Han et al., 2012). Recently, Kozlyuk et al. applied a fragment‐based approach to develop new RAGE inhibitors that can specifically directed to its ligand‐binding domain (VC1‐domain) (Kozlyuk et al., 2021). They screened binding of around 14,000 small fragments with RAGE V‐domain initially through chemical shift perturbations (CSPs) in the nuclear magnetic resonance (NMR) spectra. The lead fragments were further used to develop the X‐ray co‐crystallized structure with RAGE V and C1 domains to evaluate their exact location and orientation. In this way they identified three best binders (compounds **8–10**) that can bind to three alternative sites on RAGE receptor (Kozlyuk et al., 2021) (Figure 3).

**Figure 4:**
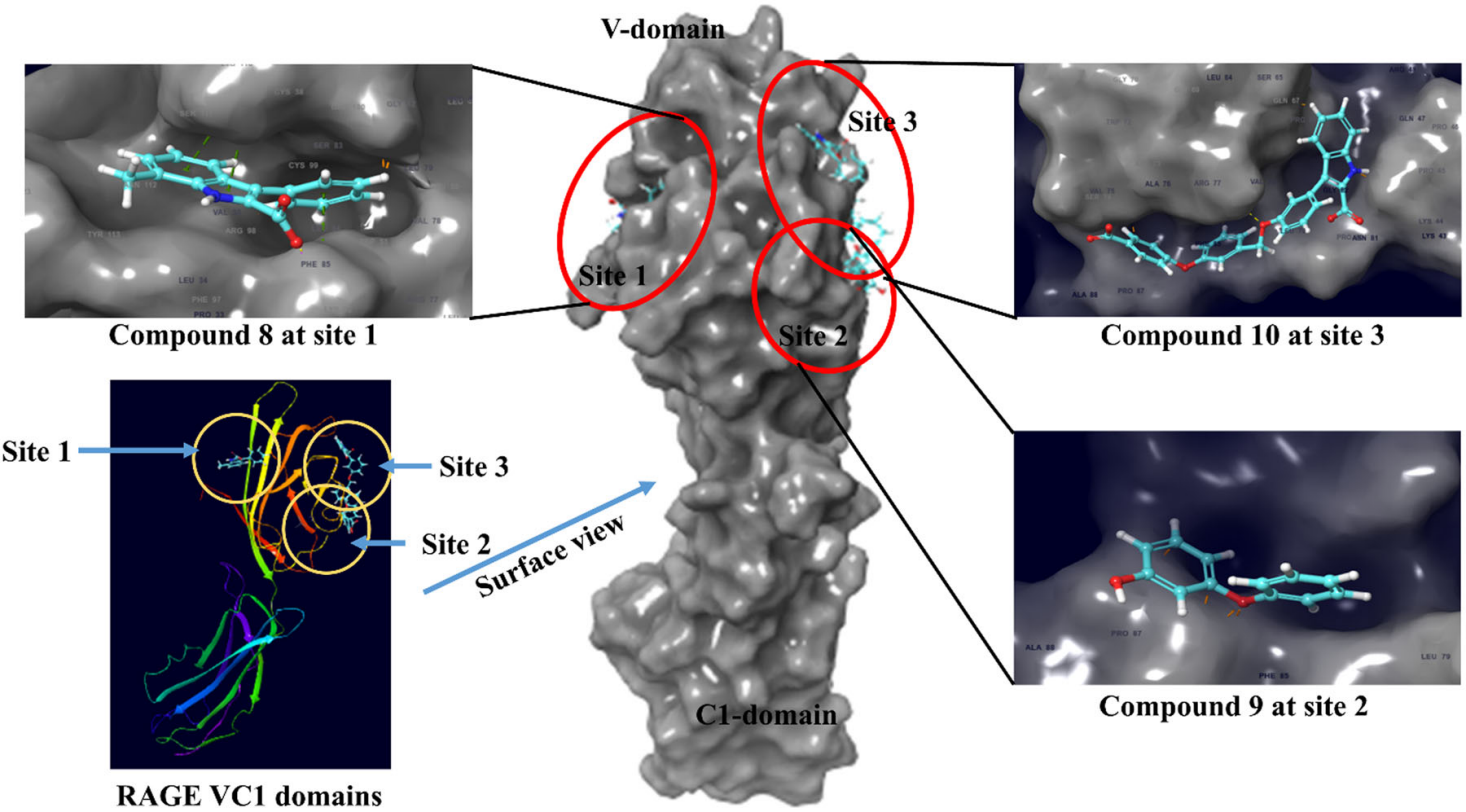
Co‐crystallized structure of RAGE VC1 domain with bound compounds **8**–**10** and their binding orientations with three different binding sites. RAGE, receptor for advanced glycation end products

Figure 4 describes the available binding sites on the RAGE receptor (extracellular domain) with their amino acid residues and orientation of compounds **8**–**10** within their specific sites. The X‐ray co‐crystallized structure of these compounds with RAGE receptor have also been published in protein data bank with PDB codes 6xq5 (binding site 1), 6xq6 (binding site 2), and 6xq3 (binding site 3) (Kozlyuk et al., 2021). To the best of our knowledge, before this study, there was no crystallographic data available of RAGE with its bound inhibitors, so it is worth schematically representing the RAGE with its bound ligands at different sites which could be useful for structure elucidation of novel RAGE binders in future studies. A major limitation of the study was the lack of data on direct inhibition of RAGE signaling. However, these results, combined with the crystal structures of three compounds, can be successfully used to further develop novel RAGE specific inhibitors.

### 3.2 Small molecule inhibitors of RAGE that can bind intracellularly

**Figure 5:**
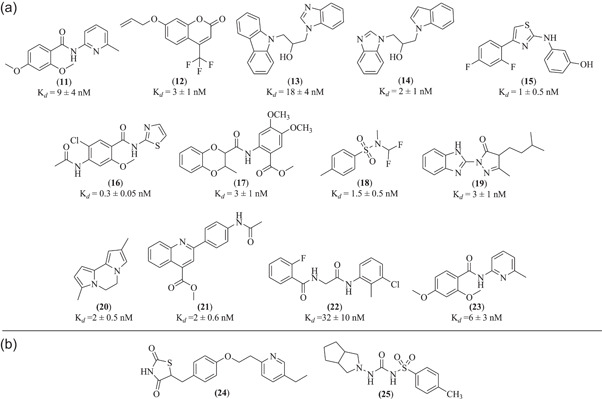
(a) Molecular structure of various RAGE inhibitors that bind to its intracellular domain; (b) Structure of RAGE inhibitors with unknown mechanism. RAGE, receptor for advanced glycation end products

The intracellular domain of RAGE is also critically responsible for various types of RAGE signaling and downstream effects. Blocking the interactions between RAGE and mammalian DIAPH1 provide therapeutic value to treat RAGE‐mediated chronic inflammatory conditions. Manigrasso et al. identified thirteen best small molecules (compounds **11**–**23**) amongst a set of 58,000 molecules that can bind to the cytoplasmic domain and inhibit the binding of RAGE and DIAPH1 (Manigrasso et al., 2016) (Figure 5a). All these compounds showed binding affinity with cytoplasmic domain of RAGE with dissociation constants ranging from 0.3 to 32 nM, among which compound **16** exhibited excellent affinity (0.3 nM) with RAGE (Figure 5a). NMR spectroscopy was performed to corroborate binding affinities with RAGE cytoplasmic domain. Careful examination of structural features of these compounds revealed that some compounds consisted of hydrophobic or aliphatic moieties with central amide linker (compounds **11**, **16**, **17**, **22**, & **23**) that may be responsible for hydrogen‐bonding interactions with RAGE cytoplasmic tail. Some compounds bearing the terminal benzimidazole moieties (compounds **13**, **14**, & **19**), the nitrogen atoms can be involved in accepting and donating the hydrogen bonds between the amino acid residues of cytoplasmic tail of RAGE (Figure 5a). RAGE‐DIAPH1‐ dependent molecular processes were significantly halted by these molecules which are mainly associated with various disease conditions such as diabetes, Alzheimer's disease, and chronic inflammation (Manigrasso et al., 2016).

### 3.3 Small molecule inhibitors of RAGE with unknown binding mechanisms

A thiazolidinone‐based drug molecule, that is, pioglitazone (compound **24**), a potent PPAR‐ℽ agonist, is also proven to be a RAGE antagonist, the administration of which significantly reduced the size of atherosclerotic plaque in diabetic ApoE^−/−^ mice (Gao et al., 2017). In high glucose treated VSMCs, compound **24** also downregulated the protein as well as mRNA expression of RAGE (Figure 5b). The mechanism of the binding of compound **24** with RAGE domains is unclear. Gliclazide (a known antidiabetic drug; compound **25**) has also been reported to abate the AGE‐RAGE pathway to treat the diabetic atherosclerosis (Jahan & Choudhary, 2021) (Figure 5b). As the study revealed gliclazide (1–100 µM) attenuated the cytokines production including IL‐1β, IL‐6, and TNF‐α in concentration‐dependent manner in RAW 264.7 macrophages and can also induce the production of anti‐inflammatory cytokines (IL‐10 and TGF‐β) (Jahan & Choudhary, 2021). However, the study does not reveal the underlying mechanism of the inhibition of AGE‐RAGE signaling by gliclazide.

## 4 LATEST DEVELOPMENT IN THE FIELD OF RAGE AND ITS INHIBITORS

RAGE is the immunoglobin family receptor which is present on most cell types and have been reported to involve in various inflammatory conditions such as cardiovascular diseases, neurodegeneration, cancer, and diabetes. RAGE can interact with several endogenous and exogenous ligands to initiate the downstream signaling that leads to chronic inflammatory conditions. Targeting RAGE and its interactions with other ligands by using various inhibitors could be a potential therapeutic treatment of these diseases. Accordingly, small inhibitors have been developed in recent years to target the extracellular and intracellular ligand binding sites of RAGE to block downstream signaling pathways. The inhibitors described in this compilation confirmed that RAGE inhibition could be a powerful approach toward the management of immune‐inflammatory diseases. The compounds in this article may act as scaffolds for further chemical refinement and optimization toward the activity, selectivity, and pharmacokinetics that could lead to future success in the management of RAGE‐associated diseases. Following the discovery and understanding of the signaling potential of RAGE, research has mainly been diverted towards the correlation between its activity and various pathological conditions including cardiovascular diseases, Alzheimer's disease, cancer, and diabetes. RAGE has become an attractive therapeutic target that can be inhibited through its extracellular and intracellular domains. So far, binding models for the interaction of RAGE with various ligands such as AGEs, S100A6, S100A11, S100A12, S100B, S100P, and DIAPH1 have been developed. However, the mechanism of binding of quinolinic acid, Aβ_1‐42_, HMGB1, TIRAP, ERK, and DOCK7 with RAGE is still unknown. Some molecules enhance the level of soluble RAGE (sRAGE) which may act as modulators of RAGE‐mediated pathways, as sRAGE has been investigated as a scavenger of its ligands to reduce their accumulation at the site of injury in organs (Emanuele et al., 2005; Falcone et al., 2005). However, sRAGE could not be an ideal therapeutic agent to target RAGE because of its large size (large recombinant protein of 36.5 kDa) and thus difficult to produce at the therapeutic level. Therefore, there is a pressing need to develop small molecule inhibitors of RAGE that could be useful in the treatment of RAGE‐associated diseases. Recently, various research groups reported small molecules that can inhibit interactions of RAGE with its ligands extracellularly or intracellularly. Such molecules may prove effective in reducing the activation of downstream signaling of RAGE to treat various disease conditions. Till 2020, three putative binding sites have been described only through molecular modeling studies or homology modeling. Later in the middle of year 2021, it was confirmed by obtaining the X‐ray co‐crystallized structures of RAGE with bound small molecules (compounds **8**–**10**). This clearly depicted three binding sites on the RAGE extracellular domain (VC1), supporting the previous molecular docking data (Kozlyuk et al., 2021) (Figure 4). NMR data have also been generated corroborating the actual binding of these molecules with RAGE extracellular domain (Kozlyuk et al., 2021). The co‐crystallized structures of RAGE with these molecules may not only lead to improve the understanding of orientation of active pockets on the extracellular domain but would also expedite the discovery of specific and potent RAGE antagonist in future. These complexes would be crucial for the generation of novel RAGE chemotypes and drug like molecules using structure‐guided drug designing approach.

## 5 CONCLUSION

Critical findings in the literature revealed that FPS‐ZM1 (compound **1**; Figure 3) still has the greatest potential in this field of research to treat various inflammatory conditions by acting as potent RAGE antagonist. FPS‐ZM1 has acceptable blood‐brain barrier permeability and disrupt RAGE‐Aβ_1‐42_ interactions with profound toxicological profile in cell culture and rodent models (Shen et al., 2017). Its Fluorine (^18^F) radiolabeled analog compound **2** (Figure 3) can potently bind to RAGE and thus used to quantify the CNS RAGE in the in vivo models (Cary et al., 2016). However, this compound still needs further in vivo characterization to demonstrate its applicability in actual clinical studies which can justify whether it could be useful in the treatment of neurological disorders or not. From the discovery of co‐crystallized structure of RAGE with bound ligand (Figure 4), it can be assumed that both compounds FPS‐ZM1 and its analog (compound **2**) can potently bind to the site 3 of RAGE VC1 domain, as indicated by their previous molecular docking data. In addition to these potent RAGE antagonists, papaverine (compound **6**; Figure 3) has also been reported to target the RAGE to further attenuate its downstream signaling through blocking the interactions of RAGE with AGEs, which could be useful in the treatment of AGE‐RAGE‐associated inflammatory disease conditions. However, comparatively higher dose of compounds **1** or **2** to inhibit these interactions could limit their clinical use as a RAGE antagonist (El‐Far et al., 2018). Besides extracellular small molecule inhibitors, molecules **11**–**23** (Figure 5a) that can target intracellular RAGE have also been reported with excellent binding affinities towards the RAGE intracellular domain (Manigrasso et al., 2016). These molecules inhibit the interactions between the intracellular ligand DIAPH1 with RAGE which can ultimately attenuate further signaling processes associated with various disease conditions. The inhibitors described in this compilation support that RAGE inhibition could be a powerful approach to the management of immune‐inflammatory diseases. A critical review of the literature suggests further investigations to look deeper into their molecular mechanism of action and to better understand the efficacy of RAGE‐targeting therapy and long‐term blockade of RAGE in humans.

## CONFLICTS OF INTEREST

The authors declare no conflicts of interest.

## DISCLOSURES

Peer reviewers on this manuscript have no relevant financial or other relationships to disclose.

## Data Availability

Not applicable; all information is gathered from published articles.
